# Sedation practices and clinical outcomes in mechanically ventilated patients in a prospective multicenter cohort

**DOI:** 10.1186/s13054-019-2394-9

**Published:** 2019-04-17

**Authors:** Romina E. Aragón, Alvaro Proaño, Nicole Mongilardi, Aldo de Ferrari, Phabiola Herrera, Rollin Roldan, Enrique Paz, Amador A. Jaymez, Eduardo Chirinos, Jose Portugal, Rocio Quispe, Roy G. Brower, William Checkley

**Affiliations:** 1 Division of Pulmonary and Critical Care, School of Medicine, Johns Hopkins University, 1830 E Monument St., Suite 555, Baltimore, MD 21287 USA; 2 Escuela Profesional de Medicina, Facultad de Medicina Alberto Hurtado, Universidad Peruana Cayetano Heredia, Lima, Peru; 3 Unidad de Conocimiento y Evidencia, Universidad Peruano Cayetano Heredia, Lima, Peru; 4 Servicio De Cuidados Intensivos, Hospital Nacional Edgardo Rebagliati Martins, Lima, Peru; 5 Servicio De Cuidados Intensivos, Hospital Nacional Guillermo Almenara Irigoyen, Lima, Peru; 6 Servicio De Cuidados Intensivos, Hospital Nacional Arzobispo Loayza, Lima, Peru; 7 Servicio De Cuidados Intensivos, Hospital De Emergencias José Casimiro Ulloa, Lima, Peru; 8 Program in Global Disease Epidemiology and Control, Department of International Health, Bloomberg School of Public Health, Johns Hopkins University, Baltimore, USA

**Keywords:** Sedation, Clinical outcomes, Critical illness

## Abstract

**Objectives:**

We sought to study the association between sedation status, medications (benzodiazepines, opioids, and antipsychotics), and clinical outcomes in a resource-limited setting.

**Design:**

A longitudinal study of critically ill participants on mechanical ventilation.

**Setting:**

Five intensive care units (ICUs) in four public hospitals in Lima, Peru.

**Patients:**

One thousand six hundred fifty-seven critically ill participants were assessed daily for sedation status during 28 days and vital status by day 90.

**Results:**

After excluding data of participants without a Richmond Agitation Sedation Scale score and without sedation, we followed 1338 (81%) participants longitudinally for 18,645 ICU days. Deep sedation was present in 98% of participants at some point of the study and in 12,942 ICU days. Deep sedation was associated with higher mortality (interquartile odds ratio (OR) = 5.42, 4.23–6.95; *p* < 0.001) and a significant decrease in ventilator (− 7.27; *p* < 0.001), ICU (− 4.38; *p* < 0.001), and hospital (− 7.00; *p* < 0.001) free days. Agitation was also associated with higher mortality (OR = 39.9, 6.53–243, *p* < 0.001). The most commonly used sedatives were opioids and benzodiazepines (9259 and 8453 patient days respectively), and the latter were associated with a 41% higher mortality in participants with a higher cumulative dose (75th vs 25th percentile, interquartile OR = 1.41, 1.12–1.77; *p* < 0.01). The overall cumulative dose of benzodiazepines and opioids was high, 774.5 mg and 16.8 g, respectively, by day 7 and by day 28; these doses approximately doubled. Haloperidol was only used in 3% of ICU days; however, the use of it was associated with a 70% lower mortality (interquartile OR = 0.3, 0.22–0.44, *p* < 0.001).

**Conclusions:**

Deep sedation, agitation, and cumulative dose of benzodiazepines were all independently associated with higher 90-day mortality. Additionally, deep sedation was associated with less ventilator-, ICU-, and hospital-free days. In contrast, haloperidol was associated with lower mortality in our study.

**Electronic supplementary material:**

The online version of this article (10.1186/s13054-019-2394-9) contains supplementary material, which is available to authorized users.

## Background

Sedation and analgesia are essential components in the care of mechanically ventilated patients in the intensive care unit (ICU) to provide comfort, improve patient-ventilator synchrony, and reduce anxiety and agitation [1]. However, deep sedation has been associated with negative patient-centered outcomes including delirium [2, 3], a common complication in the ICU, with a prevalence as high as 82% [4]. This preventable complication is an independent predictor of mortality [4–6] and is also associated with long-term cognitive impairment and disability [7–9]. Optimizing sedation practices and delirium screening with the use of protocols is associated with improved patient-centered outcomes such as a lower incidence of delirium [2, 10], fewer days on mechanical ventilation [11, 12], and an overall reduction in mortality [12, 13]. Additionally, standardized management of sedation reduces the use of sedatives without negatively affecting patient safety or increasing psychological stress [14].

Despite these recognized benefits, sedation practices remain variable, with a tendency towards over-sedation and a lack of routine delirium assessment [1, 15]. Less is known about sedation practices and delirium management for the critically ill in resource-limited settings, where mortality is higher in the USA or Europe [16]. We hypothesize that the higher mortality and longer length of stay in ICUs in resource-limited settings could be partly explained by suboptimal sedation and delirium management. In this study, we explore the association between sedation status, use of sedation and antipsychotics medications, and patient centered-outcomes in a cohort of critically ill, mechanically ventilated patients in five ICUs in Lima, Peru.

## Methods

### Study setting

A detailed description of the protocol and participating ICUs was provided elsewhere [17]. Adults with acute respiratory failure were consecutively screened in a longitudinal, observational study in five ICUs at four public hospitals in Lima, Peru. We received ethics approval from the institutional review boards at Hospital Nacional Edgardo Rebagliati Martins, Hospital Nacional Guillermo Almenara Irigoyen, Hospital Nacional Arzobispo Loayza, and Hospital de Emergencias Casimiro Ulloa in Lima, Peru, and at the Johns Hopkins School of Medicine, Baltimore, USA.

### Study design

Eligibility criteria included age ≥ 18 years, at least 24 h of invasive mechanical ventilation at one of the intensive care units participating in the study and enrollment into the study within 48 h of mechanical ventilation onset. Data collection and quality control methods were reported elsewhere [17]. At enrollment, we obtained demographics, chronic disease, and acute physiological data for all patients meeting eligibility criteria. While in the ICU, participants were followed daily to monitor use of sedation, vital status, fluid balance, clinical and ventilator management, and acute physiology during their ICU stay for either 28 days, until ICU discharge or death. Those who successfully left the ICU were followed for vital status during their inpatient hospital stay. All participants were then contacted at 90 days after enrollment to assess vital status.

### Measurements, definitions, and data collection

We collected daily cumulative dose of sedatives, neuromuscular blockers, analgesics, and antipsychotic medications. Opioid doses were converted to fentanyl equivalents, and benzodiazepines were converted to midazolam equivalents for comparison (Additional file 1: Table S1) [18, 19]. To assess the level of sedation, either the Glasgow Coma Scale [20], Ramsay Sedation Scale [21], or the Richmond Agitation Sedation Scale (RASS) [22] was used according to what was commonly applied in their ICU. To evaluate for sedation depth, we used the following scales in order of preference: RASS, Ramsay Sedation Scale, or GCS. If a participant had both RASS and another measurement for a given day, then the RASS score was used for that day. If a RASS score was not available, we then converted the Ramsay Sedation Scale or GCS to a RASS score using standardized approaches [22, 23] (Additional file 1: Table S2). We categorized sedation status as deep (≤ − 3), moderate (> − 3 but ≤ − 1), adequate (> − 1 but ≤ 1), or agitated (> 1) (Additional file 1: Table S3).

We defined ventilator-free days (VFDS) as 0 if a participant died ≤ 28 days or if mechanically ventilated for > 28 days, or as the number of days between successful weaning from mechanical ventilation and day 28 after study enrollment. Similarly, we defined ICU-free days (IFDS) as 0 if a participant died ≤ 28 days or stayed in the ICU > 28 days, or as the number of days between ICU discharge and day 28 after study enrollment. Hospital-free days (HFDS) were defined as 0 if a participant died ≤ 60 days or was hospitalized > 60 days, or as the number of days between hospital discharge and day 60 after study enrollment.

### Biostatistical methods

The main objective of this analysis was to determine the relationship between sedation status and 90-day mortality. Specifically, we evaluated sedation depth based on the RASS score, cumulative use of benzodiazepines and opioids, and use of antipsychotics in single-variable and multivariable regressions. We first constructed a multivariable logistic regression model to evaluate the association between 90-day mortality and percent of days with deep sedation or agitation, adjusted for age, sex, APACHE III, and indicator variables for ICU site. To express effect size, we used the interquartile odds ratio, i.e., the ratios of odds between the 75^th^ and 25^th^ percentiles of percent days with deep sedation or agitation. We then constructed a second multivariable logistic regression model to evaluate the association between 90-day mortality and cumulative opioids and benzodiazepines, and use of antipsychotics adjusted for the same variables as above. To express effect size, we used the interquartile odds ratio, i.e., the ratios of odds between the 75^th^ and 25^th^ percentiles of cumulative use of opioids and benzodiazepines by 28 days. We also used multivariable linear regression models to examine the above-described relationships with ventilator-free days, ICU-free days, and hospital-free days. We used R (www.r-project.org) for statistical analysis [24].

## Results

### Participant characteristics

A total of 1657 critically ill participants were enrolled in this study consisting of 21,984 ICU days. To analyze sedation depth, we excluded participants who did not receive sedatives (*n* = 294) and those without a RASS, Ramsay, or Glasgow Coma Scale score (*n* = 25). This gave us a final sample of 1338 participants (81%) followed for 18,645 ICU days. From the 1338 patients in our cohort, 869 (65%) had RASS measured at least once during their stay. From the remaining patients, 357 (27%) had at least one Ramsay measurement during their ICU stay and 112 (8%) only had been evaluated using the Glasgow Coma Scale. There were differences in age, sex, event-free days, mortality, and disease severity between participants in the final sample and those excluded (Table 1). Overall 90-day mortality was 50.1%, mean ± SD age was 58.9 ± 19.0 years, and average APACHE III score was  83.6 ± 28.3. About three quarters of admissions were for medical conditions (72.4%). None of the participating ICUs had sedation or delirium assessment tools or protocols for sedation or delirium management.

**Table 1:** Participant demographics and outcomes

Variable	Total participants (*n* = 1657)	Analytical sample (*n* = 1338)	Excluded sample (*n* = 319)	Included vs excluded *p* value
Demographics				
Male, % (*n*)	54.7 (907)	56.2 (753)	48.3 (154)	< 0.01
Age in years, mean (SD)	60.0 (18.9)	58.9 (19.0)	64.8 (17.7)	< 0.01
Main outcomes				
MV-free days, mean (SD)	10.1 (10.7)	9.6 (10.4)	12.2 (11.7)	< 0.01
ICU-free days, mean (SD)	7.3 (8.9)	6.7 (8.5)	9.9 (10.1)	< 0.01
Hospital-free days, mean (SD)	11.1 (16.6)	10.8 (16.4)	12.4 (17.5)	<0.05
90-day mortality, % (n)*	49.1 (810)	50.1 (669)	44.5 (141)	0.07
Clinical parameters				
APACHE II score, mean (SD)	24.0 (7.8)	24.1 (7.9)	23.6 (7.5)	0.22
APACHE III score, mean (SD)	82.7 (28.1)	83.6 (28.3)	78.9 (26.9)	< 0.01
SOFA score, mean (SD)	9.5 (3.5)	9.7 (3.5)	8.8 (3.6)	< 0.01
Prevalence of ARDS, % (*n*)**	17.5 (289)	19.7 (262)	8.5 (27)	< 0.01
Hospital admission type***				0.56
Medical, % (*n*)	72.3 (1196)	72.4 (968)	71.7 (228)	
Trauma, % (*n*)	10.7 (177)	11.6 (155)	6.9 (22)	
Surgical (scheduled), % (*n*)	3.5 (58)	3.1 (42)	5.0 (16)	
Surgical (unscheduled), % (*n*)	10.8 (178)	9.8 (131)	14.8 (47)	
Other, % (*n*)	2.8 (46)	3.1 (41)	1.6 (5)	

*From the 1657 participants, there were 6 participants whose death by 90 days was not recorded. Percentages (%) are obtained from all the participants whose status was known by 90 days

**From the 1657 participants there were 8 participants whose ARDS diagnosis was not determined. Percentages are obtained based on all participants where a yes/no ARDS diagnosis was recorded.

***From the 1657 participants there were 2 participants whose admission type was not obtained. Percentages are obtained from all those participants whose admission was obtained

### Patterns of sedation

**Fig. 1:**
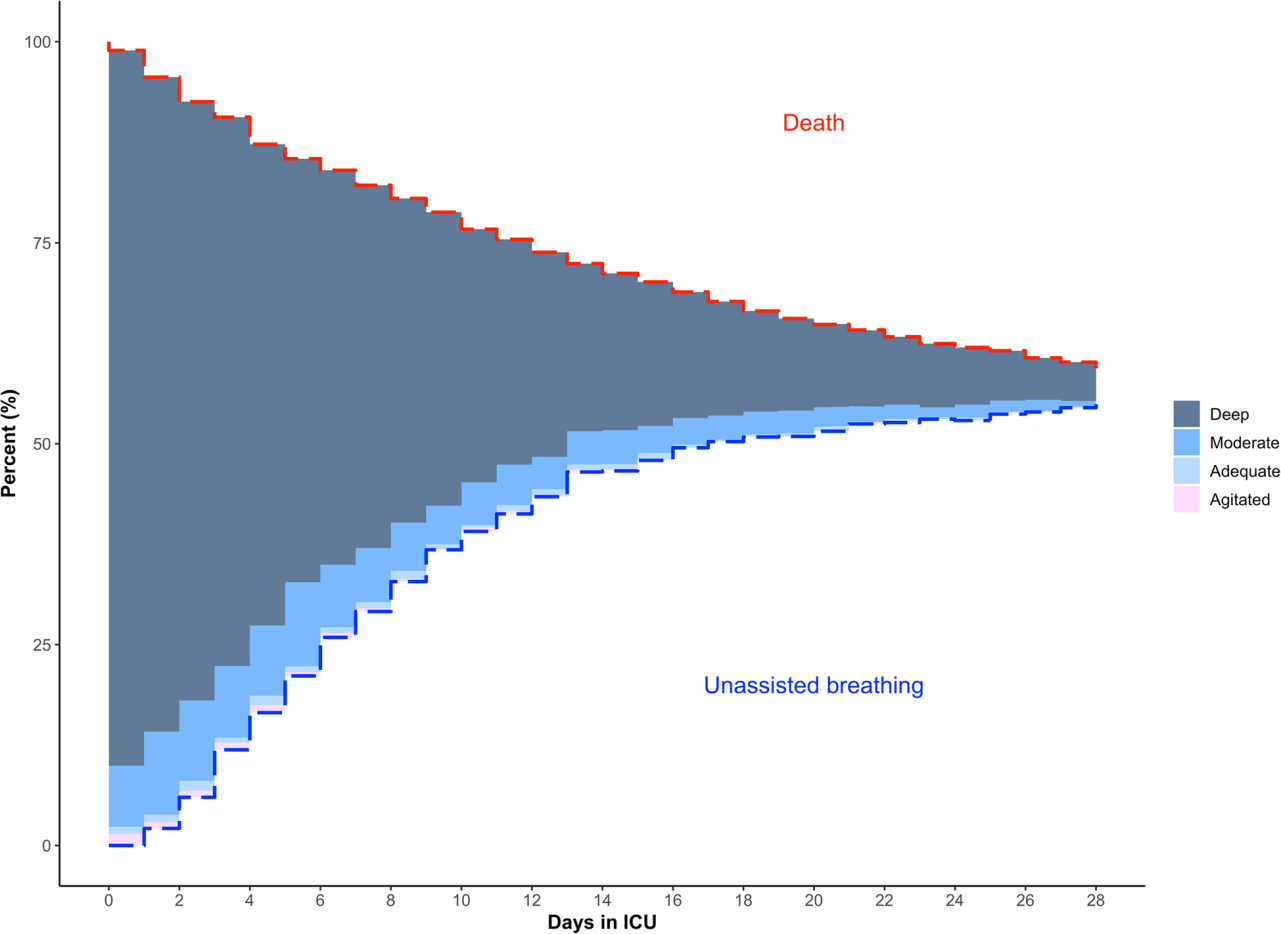
Cumulative incidence plots evaluating sedation status and vital status during ICU stay. In this figure, we plot the cumulative incidence of death (represented by a broken red line), unassisted breathing (represented by a broken blue line), and sedation status (shaded areas) among those who are receiving assisting breathing using a Berezina plot (see Additional file 1). The shaded areas were proportional to the percentage of participants who were deeply (dark blue), moderately (blue) or adequately sedated (light blue), and agitated (pink). Our categorization of sedation is based on the Richmond Agitation Sedation Scale score (RASS), and if unavailable, it is based on a conversion based on the Ramsay Scale score or the Glasgow Coma Scale score to RASS as shown in Additional file 1: Table S2. This graph excludes ICU days where a sedation score (e.g., RASS) was not recorded, giving a total number of 17,364 ICU days

**Fig. 2:**
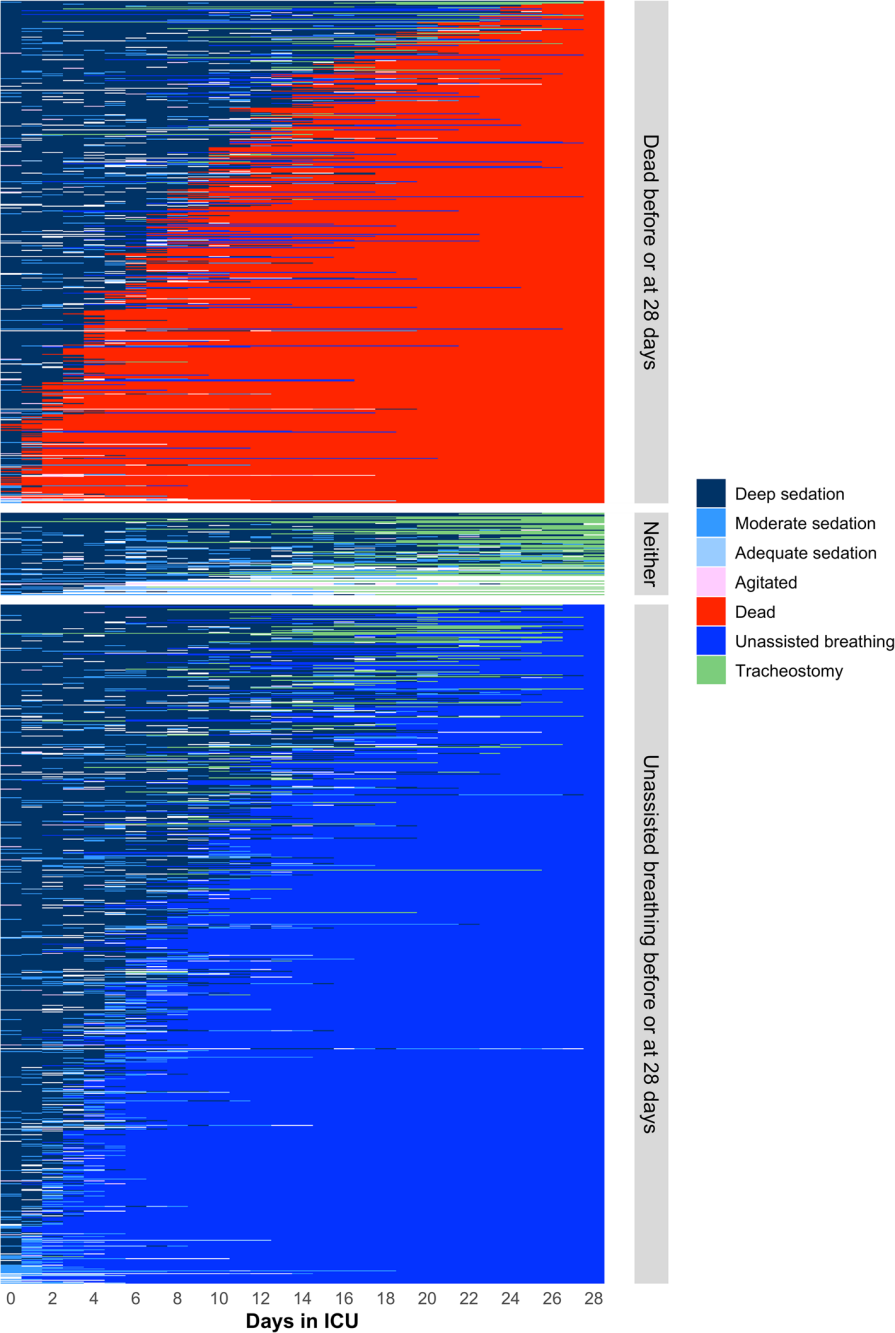
Individual-trajectory plot of sedation status, vital status, or tracheostomy status during their ICU stay. In this figure, we plot individual daily trajectories of sedation status (deep in dark blue, moderate in blue, adequate in light blue, agitated in pink), vital status (death before 28 days in red, achieved unassisted breathing by 28 days and alive in navy blue), and if the participant received a tracheostomy (in green) using a Causa plot (see Additional file 1). Our categorization of sedation is based on the Richmond Agitation Sedation Scale score (RASS), and if unavailable, it is based on a conversion based on the Ramsay Scale score or the Glasgow Coma Scale score to RASS as shown in Additional file 1: Table S2. Each row represents a study participant, and each column represents an ICU day between enrollment and day 28. We stratified the rows by vital status or if the participant was alive at 28 days but received unassisted breathing. If the patient was neither dead nor receiving unassisted breathing, they were shown as the “Neither” category by 28 days, which includes patients that were still intubated or that had a tracheostomy

**Fig. 3:**
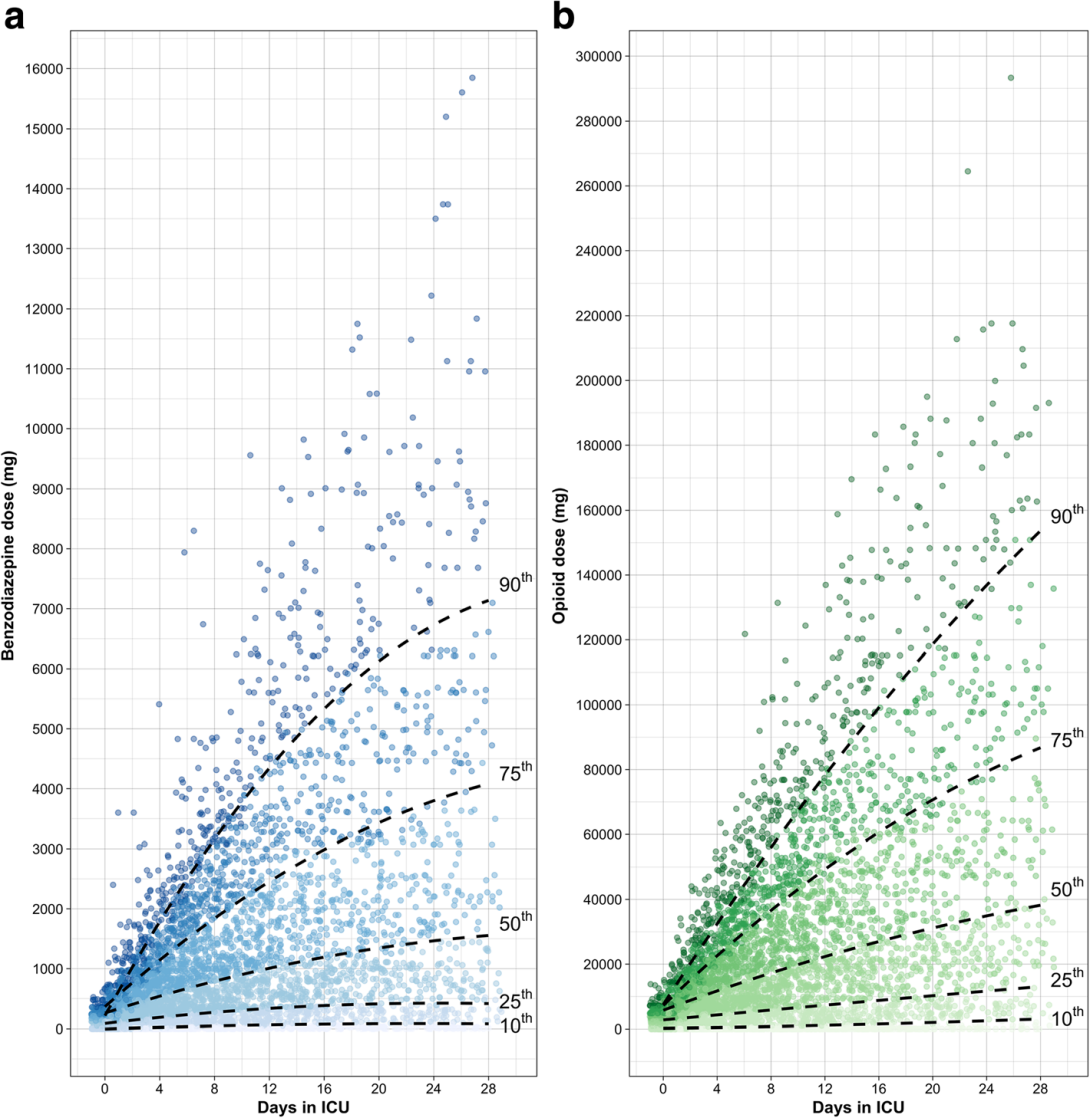
**a**, **b** Cumulative dose of pharmacological agents. We plotted individual cumulative doses of benzodiazepines (panel **a**) and opioids (panel **b**) per ICU day. Each dot represents one patient at each time point. The *x*-axis represents ICU day, and the *y*-axis the cumulative benzodiazepine or opioid dose. The broken lines represent percentiles of the cumulative doses (10^th^, 25^th^, 50^th^, 75^th^, and 90^th^ percentile, respectively)

We plotted sedation status for 28 days to describe sedation patterns (Fig. 1). Deep sedation was the most frequent level of sedation. This pattern remains even when controlling for severity (Additional file 1: Figures S1 and S2) or type of admission (medical vs surgical) (Additional file 1: Figure S3). Nearly all participants were deeply sedated during most of their ICU stay. From the 1338 participants included, 532 died and 719 achieved unassisted breathing by 28 days (Fig. 2). Of those remaining, 52 left the ICU with a tracheostomy and the other 35 were discharged from the ICU while still intubated. Moderate sedation was achieved in 62% of the patients, agitation was present in 7%, and only 26% of the patients reached an adequate sedation status at some point during the 28 days. We determined the frequency of medications administered for sedation management (Additional file 1: Table S4). The most common used sedatives were opioids and benzodiazepines, administered in about 50% of ICU days. Dexmedetomidine and propofol were rarely used, and haloperidol was only used in 3% of ICU days. The mean RASS score while receiving sedation during the 28 days in the ICU was − 3.07. The overall cumulative dose of benzodiazepines and opioids was high (Fig. 3). By day 7, median cumulative doses for benzodiazepines and opioids were 774.5 mg and 16.8 g, respectively, and by 28 days these numbers were approximately doubled.

### Sedation status and clinical outcomes

Agitation was associated with the highest risk of mortality followed by deep sedation (Table 2). The risk of mortality was five times higher for patients who spent 94% of their ICU stay in deep sedation compared to those who only spent 50% (75^th^ vs 25^th^ percentile). Secondary outcomes and their relationship with sedation status were assessed (Additional file 1: Table S5). VFDS, IFDS, and HFDS were lower with deep sedation status. In contrast, moderate sedation increased all secondary outcomes by 2 days and agitation did not have an effect in any of the secondary outcomes.

**Table 2:** Sedation status associated with 90-day mortality

Variable	Single variable	*p* value	Multivariable	*p* value
Age	1.62 (1.36–1.93)	< 0.001	1.31 (1.07–1.61)	< 0.01
Sex (males are reference)	0.94 (0.76–1.17)	0.59	0.98 (0.76–1.25)	0.86
APACHE III	2.24 (1.90–2.64)	< 0.001	1.98 (1.62–2.40)	< 0.001
% Days with deep sedation	4.70 (3.76–5.88)	< 0.001	5.42 (4.23–6.95)	< 0.001
% Days with agitation	1.57 (0.32–7.71)	0.58	39.9 (6.53–243)	< 0.001
Hospital site 1 (reference)	1.00	1.00	1.00	
Hospital site 2	0.70 (0.52–0.96)	0.03	0.63 (0.43–0.90)	0.01
Hospital site 3	0.66 (0.47–0.93)	0.02	0.79 (0.53–1.18)	0.25
Hospital site 4	0.58 (0.43–0.78)	< 0.001	0.45 (0.32–0.62)	< 0.001
Hospital site 5	1.35 (0.89–2.06)	0.16	0.93 (0.57–1.54)	0.78

### Sedation use and clinical outcomes

We evaluated the cumulative dose of benzodiazepines and opioids, adjusting for variables associated with mortality (Table 3). Benzodiazepines but not opioids were associated with higher risk of mortality. The higher cumulative dose of benzodiazepines (75^th^ vs 25^th^ percentile) was associated with a 41% higher mortality. Additionally, the use of antipsychotics was associated with lower mortality by nearly 70%.

**Table 3:** Pharmacological agents associated with 90-day mortality

Variable	Unadjusted model	*p* value	Adjusted model	*p* value
Age	1.62 (1.36–1.93)	< 0.001	1.34 (1.10–1.62)	< 0.001
Sex (males are reference)	0.94 (0.76–1.17)	0.59	0.93 (0.74–1.18)	0.55
APACHE III	2.24 (1.90–2.64)	< 0.001	2.17 (1.80–2.60)	< 0.001
Use of antipsychotics	0.31 (0.23–0.42)	< 0.001	0.31 (0.22–0.44)	< 0.001
Cumulative dose of benzodiazepines	1.07 (0.98–1.16)	0.12	1.41 (1.12–1.77)	< 0.01
Cumulative dose of opioids	1.01 (0.93–1.10)	0.77	0.82 (0.64–1.05)	0.12
Hospital site 1 (reference)	1.00	1.00	1.00	
Hospital site 2	0.70 (0.52–0.96)	0.03	0.63 (0.45–0.89)	0.01
Hospital site 3	0.66 (0.47–0.93)	0.02	0.84 (0.58–1.22)	0.36
Hospital site 4	0.58 (0.43–0.78)	< 0.001	0.74 (0.54–1.01)	0.06
Hospital site 5	1.35 (0.89–2.06)	0.16	0.61 (0.38–0.99)	0.04

## Discussion

We found that deep sedation, agitation, and benzodiazepines were independently associated with worse clinical outcomes. Specifically, a greater percentage of days spent in deep sedation (i.e., 75^th^ vs 25^th^ percentile of days in deep sedation) was associated with a fivefold greater odds of mortality and a 4- to 7-point reduction in ventilator-free, ICU-free, and hospital-free days. Agitation status had a 40-fold higher mortality. The interquartile cumulative difference in benzodiazepine usage was associated with a 41% higher odds of 90-day mortality. Additionally, we reported that the usage of antipsychotics was associated with lower 90-day mortality. We identified that most of our critically ill participants undergoing mechanical ventilation were deeply sedated throughout their ICU stay. The most common used sedatives were opioids and benzodiazepines.

Our findings confirm the known relationship between sedation depth and use of benzodiazepines with adverse outcomes. Our results regarding the association between deep sedation and mortality as well as deep sedation and decrease in the secondary outcomes are consistent with previous similar studies [25, 26]. However, in our cohort of patients, significant variation of sedation depth as reported by Shehabi et al. [25] is not present. Unfortunately, most of our enrolled participants remained deeply sedated past the first 48 h after initiation of mechanical ventilation.

In our study, we identified an independent relationship between benzodiazepines and mortality. Previous studies support the current recommendations of non-benzodiazepine agents [27, 28]. In a recent meta-analysis by Fraser et al. that included six trials enrolling 1235 critically ill participants, the use of non-benzodiazepine sedation in medical and surgical adult ICU patients was not associated with a statistically significant increase in mortality but was associated with 1.65-day shorter length of ICU stay and 1.9-day shorter duration of mechanical ventilation compared to patients receiving benzodiazepines for sedation [29].

None of the five ICUs participating in this study used protocols for sedation management, nor did they use tools to screen or manage delirium [17]. This is not surprising since data from previous international surveys reported implementation rates between 20% and 80% [1], including a study of 912 ICU practitioners in high-income countries that revealed that only 16% used a valid delirium assessment tool [30]. We show that physicians in Peruvian ICUs mainly use benzodiazepines and opioids, and the use of dexmedetomidine is still limited. One of the reasons for the low usage of dexmedetomidine could be its high price; however, when considering the potential benefits, it is possible that it may actually be more cost-effective than using benzodiazepines [31].

Notably, our study showed that the use of haloperidol was associated with a lower mortality in ICU patients. Even though we did not assess delirium directly in our patients, we used haloperidol as a surrogate for the treatment of ICU delirium. Previous evidence shows that the use of antipsychotics could reduce the incidence of delirium [32]. The effect of delirium management with antipsychotic medications on mortality in critically ill patients is unknown, and adequately powered randomized control trials are needed. However, a recent randomized controlled trial that used haloperidol or ziprasidone, as compared with placebo, in patients with acute respiratory failure or shock and hypoactive or hyperactive delirium in the ICU did not find a reduction in secondary outcomes such as 30-day or 90-day mortality [33].

There are important limitations in this study. First, we did not evaluate for delirium. However, evaluation of delirium was not an aim of the primary study and participating ICUs did not use delirium screening scales. Nonetheless, assessment of delirium using the CAM-ICU [34] or another validated survey would have provided a better understanding of the magnitude of the problem, given that delirium is a well-recognized factor that affects sedation practices [35]. Second, the assessment of sedation depth was conducted with non-standard instruments like the Glasgow Coma Scale [20]. Nevertheless, the Glasgow Coma Scale has a strong correlation with RASS Sedation Scale [22]. Still, given the design of the Glasgow Coma Scale, agitation status could have been underestimated. Another limitation is that we did not take into account the primary pathology when evaluating sedation practices. Primary strengths of this study are that it is a large prospective multicenter assessment of routine practices in a broad range of critically ill patients undergoing mechanical ventilation. Additionally, it provides detailed data about sedation practices and their impact on patient outcomes throughout the ICU stay in a middle-income country, which is very important for generalizing previous findings from high-income settings. Another important strength resides in the high quality of our data, assured by a tiered approached quality control of the report forms, double-data entry, and a centrally coordinated database.

## Conclusions

The results of our study indicate that despite strong evidence that correlates sedation depth with worse clinical outcomes, most ICU patients were deeply sedated during their ICU stay. This high level of sedation could potentially account for the high mortality observed in our patient population and warrants timely sustainable implementation strategies that apply to low- and middle-income countries, such as standardized protocols. However, future studies should also evaluate sedation depth and mortality adjusted for newer severity scoring systems in the ICU (e.g., APACHE IV). This way, the adverse outcomes related to these preventable measures can be avoided.

## Additional file

Additional file 1: **Table S1.** Dose Equivalency of Benzodiazepines Relative to Midazolam and Opioids relative to Fentanyl. **Table S2.** Ramsay Sedation and Glasgow Coma Scales conversion to the Richmond Agitation-Sedation Scale. **Table S3.** Sedation Status corresponding to Richmond Agitation-Sedation score. **Table S4.** Sedation Status and Use of Sedatives, Antipsychotics and Neuromuscular Blockers. **Table S5.** Sedation Status and Secondary Outcomes. **Figure S1.** Cumulative Incidence Plots Evaluating Sedation Status and Vital Status during ICU Stay stratified by ARDS Status at Enrollment. We plot the cumulative incidence of death (broken red line), unassisted breathing (broken blue line), and sedation status (shaded areas) among those who are receiving assisting breathing, stratified by whether participants have ARDS on admission (panel A) or no ARDS (panel B). The shaded areas were proportional to the percentage of participants who were deeply (dark blue), moderately (blue) or adequately sedated (light blue), and agitated (pink). Based on the Richmond Agitation Sedation Scale score or its conversion **Figure S2.** Cumulative Incidence Plots Evaluating Sedation Status and Vital Status during ICU Stay Stratified by APACHE III Score at Enrollment. We plot the cumulative incidence of death (broken red line), unassisted breathing (broken blue line), and sedation status (shaded areas) among those who are receiving assisting breathing, stratified by APACHE III score (panel A: 0–69, panel B: 70–96, panel C: 97–179). Shaded areas and categorization of sedation same as in Figure S1. **Figure S3.** Cumulative Incidence Plots Evaluating Sedation Status and Vital Status during ICU Stay Stratified by Admission Type. We plot the cumulative incidence of death (broken red line), unassisted breathing (blue line), and sedation status (shaded areas) among those who are receiving assisting breathing, stratified by whether admission was medical (panel A) or surgical (panel B). Shaded areas and categorization of sedation same as in Figure S1. (PDF 1953 kb)
