# Supplementary material for: Sedation practices and clinical outcomes in mechanically ventilated patients in a prospective multicenter cohort
Source: Crit Care. 2019 Apr 17;23:130. doi: 10.1186/s13054-019-2394-9 (PMC6472077; doi:10.1186/s13054-019-2394-9)
Supplement: Supplementary file 1 — Table S1. Dose Equivalency of Benzodiazepines Relative to Midazolam and Opioids relative to Fentanyl. Table S2. Ramsay Sedation and Glasgow Coma Scales conversion to the Richmond Agitation-Sedation Scale. Table S3. Sedation Status corresponding to Richmond Agitation-Sedation score. Table S4. Sedation Status and Use of Sedatives, Antipsychotics and Neuromuscular Blockers. Table S5. Sedation Status and Secondary Outcomes. Figure S1. Cumulative Incidence Plots Evaluating Sedation Status and Vital Status during ICU Stay stratified by ARDS Status at Enrollment. We plot the cumulative incidence of death (broken red line), unassisted breathing (broken blue line), and sedation status (shaded areas) among those who are receiving assisting breathing, stratified by whether participants have ARDS on admission (panel A) or no ARDS (panel B). The shaded areas were proportional to the percentage of participants who were deeply (dark blue), moderately (blue) or adequately sedated (light blue), and agitated (pink). Based on the Richmond Agitation Sedation Scale score or its conversion Figure S2. Cumulative Incidence Plots Evaluating Sedation Status and Vital Status during ICU Stay Stratified by APACHE III Score at Enrollment. We plot the cumulative incidence of death (broken red line), unassisted breathing (broken blue line), and sedation status (shaded areas) among those who are receiving assisting breathing, stratified by APACHE III score (panel A: 0–69, panel B: 70–96, panel C: 97–179). Shaded areas and categorization of sedation same as in Figure S1. Figure S3. Cumulative Incidence Plots Evaluating Sedation Status and Vital Status during ICU Stay Stratified by Admission Type. We plot the cumulative incidence of death (broken red line), unassisted breathing (blue line), and sedation status (shaded areas) among those who are receiving assisting breathing, stratified by whether admission was medical (panel A) or surgical (panel B). Shaded areas and categorization of sedation same a [file 13054_2019_2394_MOESM1_ESM.pdf]

ONLINE SUPPLEMENT FOR "Sedation Practices and Clinical Outcomes in Mechanically Ventilated Patients in a Prospective Multicenter Cohort in Peru"

Romina E. Aragón, Alvaro Proaño, Nicole Mongilardi, Aldo de Ferrari, Phabiola Herrera, Rollin Roldan, Enrique Paz, Amador A. Jaymez, Eduardo Chirinos, Jose Portugal, Rocio Quispe, Roy G. Brower, William Checkley.

Pharmacological Equivalence

For benzodiazepines we converted diazepam and lorazepam to its equivalent midazolam, as converted previously in the literature [1]. Based on literature values we also converted morphine, meperidine, remifentanil to fentanyl [1, 2]. This helped us evaluate the cumulative dose of benzodiazepines and opioids, based on midazolam and fentanyl, respectively (E-Table 1).

Score Conversions

We chose Richmond Agitation-Sedation Scale (RASS) as the standard, since is the most valid and reliable tool for measuring quality and depth of sedation in adult intensive care unit (ICU) patients [3]. We constructed an algorithm to convert Glasgow Coma Scale (GCS) or Ramsay Sedation Scale to RASS (E-Table 2), for those participants without registered RASS score based on prior data [4, 5]. If both scales were available for a patient, then we used the Ramsay Sedation Scale since it was created to evaluate sedation status. Once we obtained our new converted RASS score, we divided them into four categories, as shown in E-Table 3.

Patient-Days Characteristics in the 1,338 Study Participants

In E-Table 4, we show the number of patient-days that were evaluated, and how RASS scores and pharmacological agents were used within the study.

The Berezina Plot

We wrote all our graphics using ggplot2 package [6], in R software [7]. Our first graph, which we call the Berezina graph – inspired by the Berezina Battle of 1812 – is the applied form of a theoretical graph from our group [8]. In this graph we explore sedation status, while accounting for how many patients stopped requiring assisted breathing and those patients that died during their stay in the ICU.
We evaluated our plots in different subpopulations from our final sample study. We categorized patients as having or not ARDS on admission. Their sedation status pattern throughout time was similar, as seen below.
For others to recreate our graph, we are providing our code here. Please take into account that we also use the package reshape and data.table, which will help us manipulate our data appropriately. Here we have three important variables: id, dayn and depth. ID is the subject's identifier in the study, dayn is the day of the study and depth is what we are evaluating which can be either adequate, agitated, dead, deep, discharged or moderate.

```
library(ggplot2)
library(reshape2)
library(data.table)
dt_data <- data.table(toty)
dt_long <- melt( dt_data, id.vars = c("id","dayn"),
measure.vars = c("depth"))
dt_long <- dt_long[ !is.na(value)]
k_len <- nrow(dt_long)
dt_long[ , num_per_day:=length(value), by=dayn]
dt_agg <- dt_long[, .(perc= .N/unique(num_per_day)*100 ),
```

```
by= .(dayn, value)]
```

When doing this, we should get something similar to this:

```
head(dt_agg)
   dayn     value      perc
1:    0      Deep 90.133125
2:    1      Deep 86.385737
3:    2      Deep 82.124789
```

We advise other researchers to curate their data, every day of the study must have a value, so if it's not present, we advise to do the following, to curate:

```
alv1 <- list(0,"Dead",0)
alv2 <- list(0,"Discharged",0)
alv3 <- list(16,"Agitated",0)
alv4 <- list(17,"Agitated",0)
alv5 <- list(19,"Agitated",0)
alv6 <- list(23,"Agitated",0)
alv7 <- list(26,"Agitated",0)
alv8 <- list(27,"Agitated",0)
alv9 <- list(28,"Adequate",0)
dt_agg1<-
rbind(dt_agg,alv1,alv2,alv3,alv4,alv5,alv6,alv7,alv8,alv9)
dt_agg1$value = factor(dt_agg1$value,levels =
c("Discharged","Agitated","Adequate","Light","Deep","Dead")
)
```

Then we need to transform, and will be ready to create our graph.

```
df2_2 <- rbind(dt_agg1,
        transform(dt_agg1[order(dt_agg1$dayn),],
                  dayn=dayn - 1e-9,  # required to avoid
crazy steps
                  perc=ave(perc, value, FUN=function(z)
c(z[[1]], head(z, -1L)))))
setorder(df2_2, dayn, value)
df2_2$value = factor(df2_2$value, levels =
rev(levels(df2_2$value)))
g1a<-ggplot(df2_2, aes(x=dayn, y=perc, fill=value)) +
geom_area() + scale_fill_manual(name="",labels = c("
","Deep","Moderate","Adequate","Agitated","
"),values=alpha(c("white", "#003366",
"#3399FF","#99CCFF","#FFCCFF","white"),0.7))
dt_aggDis = df2_2[df2_2$value=="Discharged"]
g1b<-g1a+geom_step(data=dt_aggDis,colour='blue',linetype =
"longdash",size=1)
dt_aggDe = df2_2[df2_2$value=="Dead"]
dt_aggDe$perc = 100-dt_aggDe$perc
g1c<-g1b+geom_step(data=dt_aggDe,colour='red',linetype =
"longdash",size=1)
g1d<-g1c+ylab("%")+xlab("Days in
ICU")+scale_x_continuous(breaks=pretty_breaks(n=12))
g1e<-g1d+annotate("text", x = 20, y = 90, label = "Death",
colour="red")+annotate("text", x = 20, y = 20, label =
"Unassisted breathing", colour="blue")+theme_classic()
```

The Causa Plot

Similar to lasagna plots [9], we have created a heatmap-based graph that helps to evaluate longitudinal data with an important critical care outcome: death or extubation by 28 days. Unlike the Berezina plot, this is a subject-specific graph. However, our plot does not use the same code as our Lasagna colleagues, but instead we like to refer to a different way of writing graphs [10], which is implemented in ggplot2 [6].

```
toty_total2 =
toty_total[c("id","dayn","depth","deep.sum","state")]
```

Where id is the id of each subject, dayn is the day of the study, deep.sum is how many days were considered “deep”, based on our previous definition. State defines if by 28 days the patient was extubated, died, or neither.

```
library(ggplot2)
ggplot(toty_total2,aes(x=dayn,y=reorder(factor(id),deep.sum
),fill=factor(depth))) + geom_tile(colour='black')+
theme(axis.ticks = element_blank(), axis.text.y =
element_blank())+facet_grid(state~.,scales="free") +
xlab("Days in ICU")+ylab("1327
Partcipants")+scale_fill_manual(name="",values=(c('red',
"#003366","#3399FF","#99CCFF","#FFCCFF",'blue')))+scale_x_c
ontinuous(breaks=pretty_breaks(n=20)) +
theme(legend.position="top")
```

We like the simplicity of our plot, which given its distribution it reminds us of Peruvian dish: *causa limeña*. We recommend using this simple code to re-create and evaluate their ICU data or any other longitudinal data.

Secondary Outcomes

By using our secondary outcomes (ventilator free days, ICU free days, hospital free days) we constructed single and multivariable regressions. In E-Table 5, we present the difference between the 75th and the 25th percentile of each variable. This model was adjusted for age, sex, APACHE III and usage or not of antipsychotics, using linear regression.

References

1. Wilson WC, Smedira NG, Fink C, McDowell JA, Luce JM: **Ordering and administration of sedatives and analgesics during the withholding and withdrawal of life support from critically ill patients**. *JAMA* 1992, **267**(7):949-953.
2. Lang E, Kapila A, Shlugman D, Hoke JF, Sebel PS, Glass PS: **Reduction of isoflurane minimal alveolar concentration by remifentanil**. *Anesthesiology* 1996, **85**(4):721-728.
3. Barr J, Fraser GL, Puntillo K, Ely EW, Gelinas C, Dasta JF, Davidson JE, Devlin JW, Kress JP, Joffe AM *et al*: **Clinical practice guidelines for the management of pain, agitation, and delirium in adult patients in the intensive care unit**. *Crit Care Med* 2013, **41**(1):263-306.
4. Sessler CN, Gosnell MS, Grap MJ, Brophy GM, O'Neal PV, Keane KA, Tesoro EP, Elswick RK: **The Richmond Agitation-Sedation Scale: validity and reliability in adult intensive care unit patients**. *Am J Respir Crit Care Med* 2002, **166**(10):1338-1344.
5. Ely EW, Truman B, Shintani A, Thomason JW, Wheeler AP, Gordon S, Francis J, Speroff T, Gautam S, Margolin R *et al*: **Monitoring sedation status over time in ICU patients: reliability and validity of the Richmond Agitation-Sedation Scale (RASS)**. *JAMA* 2003, **289**(22):2983-2991.
6. Wickham H: **ggplot2: Elegant Graphics for Data Analysis**: Springer-Verlag New York; 2016.
7. R Core Team: **R: A language and environment for statistical computing. R Foundation for Statistical Computing**. In. Vienna, Austria. ; 2016.
8. Checkley W, Brower RG, Munoz A, Investigators NIHARDSN: **Inference for mutually exclusive competing events through a mixture of generalized gamma distributions**. *Epidemiology* 2010, **21**(4):557-565.
9. Swihart BJ, Caffo B, James BD, Strand M, Schwartz BS, Punjabi NM: **Lasagna plots: a saucy alternative to spaghetti plots**. *Epidemiology* 2010, **21**(5):621-625.
10. Wilkinson L: **The Grammar of Graphics (Statistics and Computing)**: Springer-Verlag; 2005.

Table S1 Dose Equivalency of Benzodiazepines Relative to Midazolam and Opioids Relative to Fentanyl.

Benzodiazepine agent	Equivalent to 2.5 mg of Midazolam
Diazepam	5 mg
Lorazepam	1 mg
**Opioid agent**	**Equivalent to 0.15 mg of Fentanyl**
Morphine	10 mg
Meperidine	75 mg
Remifentanil	0.125 mg

Table S2 Ramsay Sedation and Glasgow Coma Scales conversion to the Richmond Agitation-Sedation Scale.

Glasgow Coma Scale	Richmond Agitation-Sedation Scale	Ramsay Sedation Scale
	2	1
	1	
15	0	2 to 3
14	-1	
12 to 13	-2	4
9 to 11	-3	
4 to 7	-4	5 to 6
3	-5	

Table S3 Sedation Status Corresponding to Richmond Agitation-Sedation Score.

Sedation Status	Richmond Agitation-Sedation Score
Agitated	>1
Adequate sedation	>-1 but ≤1
Moderate sedation	>-3 but ≤-1
Deep sedation	≤-3

Table S4 Sedation Status and Use of Sedatives, Antipsychotics and Neuromuscular Blockers.

	Days Evaluated			
**Variable**	0-28	0-2	3-7	8-28
Number of Patient-Days Assessed	18,645	3,910	5,429	9,306
RASS score, mean	-3.07	-3.67	-3.16	-2.76
RASS score, median	-3	-4	-3	-3
Benzodiazepines	8,453	2,906	2,580	2,967
Opioids	9,259	3,101	2,942	3,216
Dexmedetomidine	114	15	27	72
Propofol	2	0	0	2
Barbiturates	135	35	53	47
Neuromuscular Blockers	62	14	21	27
Haloperidol	483	45	157	281

Table S5 Sedation Status and Secondary Outcomes.

Secondary Outcome	Sedation Status (% Days in ICU)	Single Variable (75th - 25th)	p-value	Multivariable (75th - 25th)	p-value
Ventilator Free Days	Deep Sedation	-8.73	<0.001	-7.27	<0.001
	Agitated	0	0.88	0	<0.01
	Moderate Sedation	6.29	<0.001	1.41	<0.01
ICU Free Days	Deep Sedation	-6.02	<0.001	-4.38	<0.001
	Agitated	0	0.83	0	0.06
	Moderate Sedation	4.60	<0.01	1.68	<0.001
Hospital Free Days	Deep Sedation	-9.41	<0.01	-7.00	<0.001
	Agitated	0	0.88	0	<0.05
	Moderate Sedation	7.20	<0.01	2.06	0.02

Figure S1 Cumulative Incidence Plots Evaluating Sedation Status and Vital Status During ICU Stay stratified by ARDS Status at Enrollment. In this figure, we plot the cumulative incidence of death (represented by a broken red line), unassisted breathing (represented by a broken blue line), and sedation status (shaded areas) among those who are receiving assisting breathing, stratified by whether participants have ARDS on admission (left panel) or no ARDS (right panel). The shaded areas were proportional to the percentage of participants who were deeply (dark blue), moderately (blue) or adequately sedated (light blue), and agitated (pink). Our categorization of sedation is based on the Richmond Agitation Sedation Scale score (RASS), and if unavailable it is based on a conversion based on the Ramsay Scale score or the Glasgow Coma Scale score to RASS as shown in Additional file 1: Table S2.

Figure S2 Cumulative Incidence Plots Evaluating Sedation Status and Vital Status During ICU Stay Stratified by APACHE III Score at Enrollment. In this figure, we plot the cumulative incidence of death (represented by a broken red line), unassisted breathing (represented by a broken blue line), and sedation status (shaded areas) among those who are receiving assisting breathing, stratified by APACHE III score (0–69, 70–96, 97–179). The shaded areas were proportional to the percentage of participants who were deeply (dark blue), moderately (blue) or adequately sedated (light blue), and agitated (pink). Finally, we plotted this graph according to their admission type and found a similar sedation depth pattern. Our categorization of sedation is based on the Richmond Agitation Sedation Scale score (RASS), and if unavailable it is based on a conversion based on the Ramsay Scale score or the Glasgow Coma Scale score to RASS as shown in Additional file 1: Table S2.

Figure S3 Cumulative Incidence Plots Evaluating Sedation Status and Vital Status During ICU Stay Stratified by Admission Type. In this figure, we plot the cumulative incidence of death (represented by a broken red line), unassisted breathing (represented by a broken blue line), and sedation status (shaded areas) among those who are receiving assisting breathing, stratified by whether admission was medical (left) or surgical (right). The shaded areas were proportional to the percentage of participants who were deeply (dark blue), moderately (blue) or adequately sedated (light blue), and agitated (pink). Our categorization of sedation is based on the Richmond Agitation Sedation Scale score (RASS), and if unavailable it is based on a conversion based on the Ramsay Scale score or the Glasgow Coma Scale score to RASS as shown in Additional file 1: Table S2.

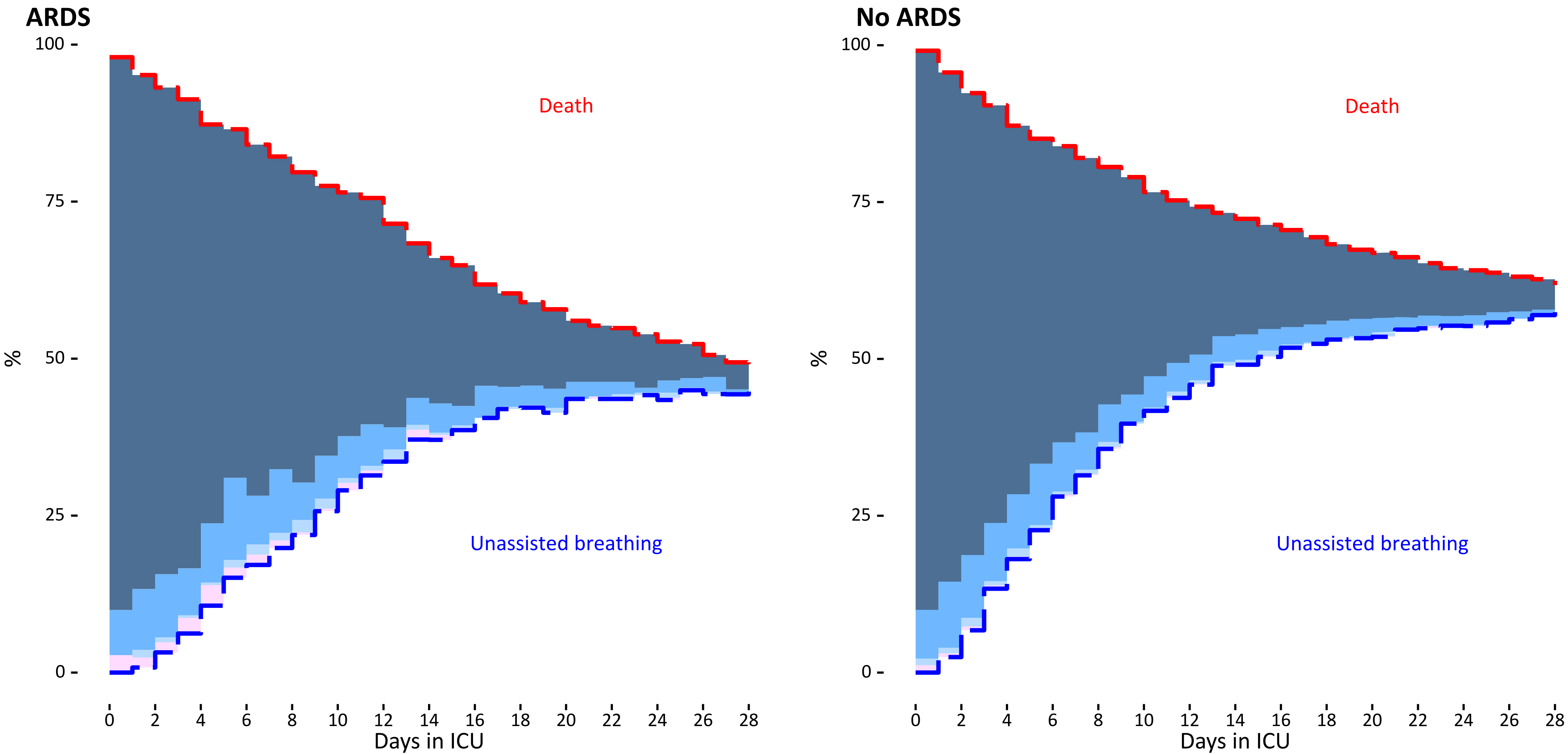
ARDS
100
75
50
25
0
%
Death
Unassisted breathing
0 2 4 6 8 10 12 14 16 18 20 22 24 26 28
Days in ICU
No ARDS
100
75
50
25
0
%
Death
Unassisted breathing
0 2 4 6 8 10 12 14 16 18 20 22 24 26 28
Days in ICU

Figure S1

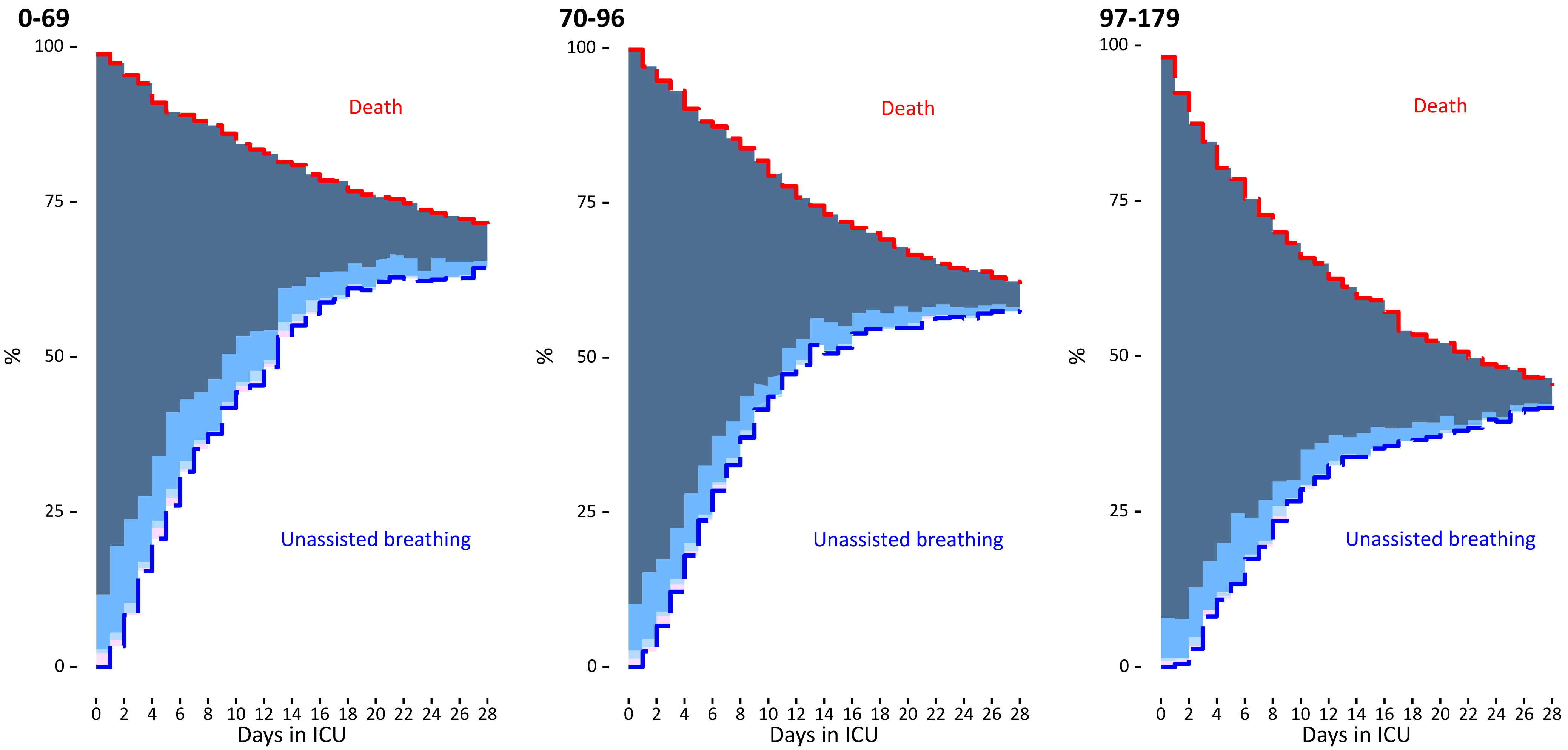
0-69
70-96
97-179
100
75
50
25
0
%
Death
Unassisted breathing
0 2 4 6 8 10 12 14 16 18 20 22 24 26 28
Days in ICU

Figure S2

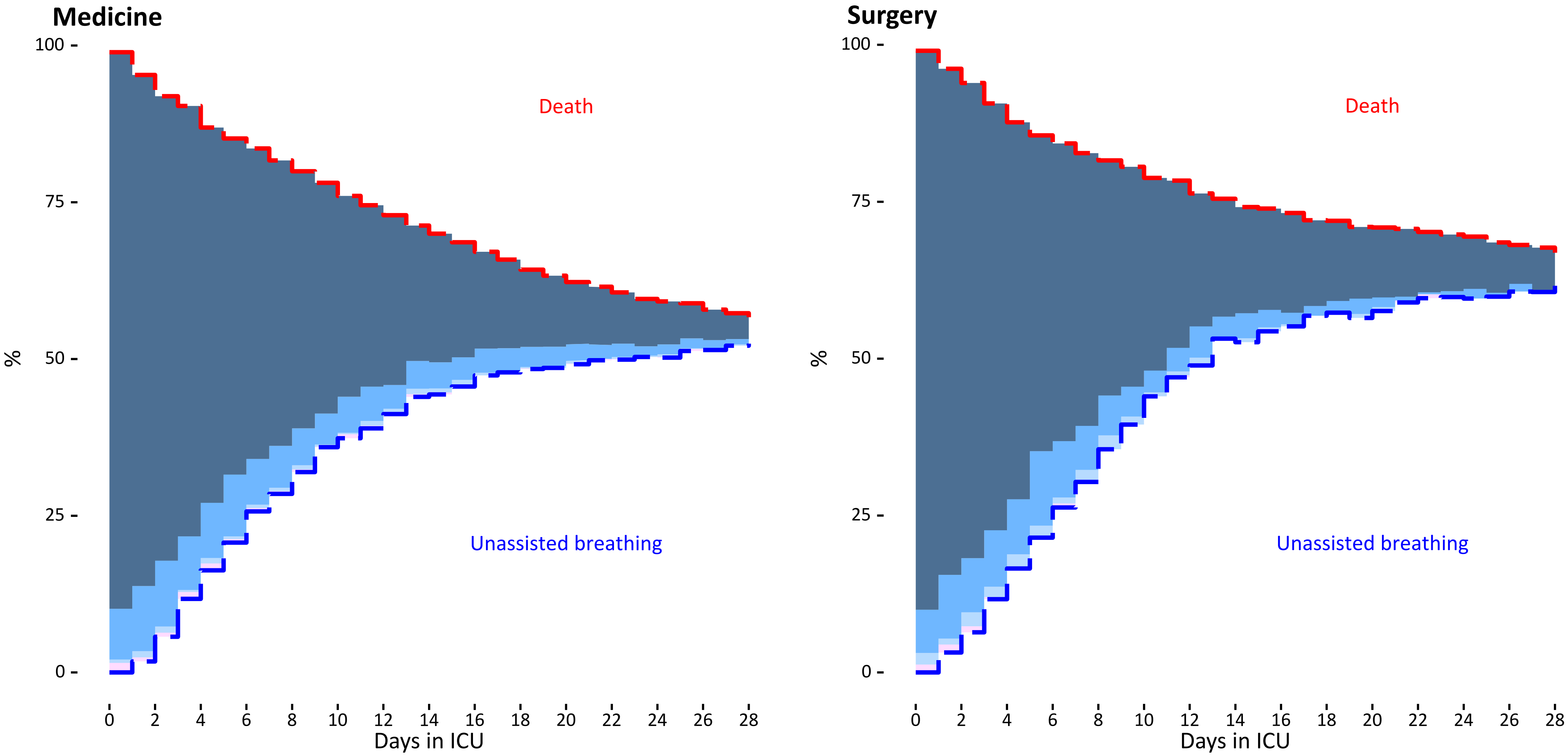
Medicine
100
75
50
25
0
%
Death
Unassisted breathing
0 2 4 6 8 10 12 14 16 18 20 22 24 26 28
Days in ICU
Surgery
100
75
50
25
0
%
Death
Unassisted breathing
0 2 4 6 8 10 12 14 16 18 20 22 24 26 28
Days in ICU

Figure S3
